# Determinants of Kidney Function and Accuracy of Kidney Microcysts Detection in Patients Treated With Lithium Salts for Bipolar Disorder

**DOI:** 10.3389/fphar.2021.784298

**Published:** 2022-01-07

**Authors:** Nahid Tabibzadeh, Anne-Laure Faucon, Emmanuelle Vidal-Petiot, Fidéline Serrano, Lisa Males, Pedro Fernandez, Antoine Khalil, François Rouzet, Coralie Tardivon, Nicolas Mazer, Caroline Dubertret, Marine Delavest, Emeline Marlinge, Bruno Etain, Frank Bellivier, François Vrtovsnik, Martin Flamant

**Affiliations:** ^1^ Physiologie Rénale-Explorations Fonctionnelles, FHU APOLLO, Assistance Publique Hôpitaux de Paris, Hôpital Bichat-Claude Bernard, Paris, France; ^2^ Centre de Recherche des Cordeliers, INSERM, Sorbonne Université, Université de Paris, Laboratoire de Physiologie Rénale et Tubulopathies, F-75006, Paris, France; ^3^ CNRS ERL 8228–Unité Métabolisme et Physiologie Rénale, F-75006, Paris, France; ^4^ Centre de recherche en Epidémiologie et Santé des Populations, INSERM UMR 1018, Renal and Cardiovascular Epidemiology, Université Paris-Saclay, Paris, France; ^5^ Université de Paris, Paris, France; ^6^ Inserm U1149, Paris, France; ^7^ UF d’Hormonologie, Assistance Publique Hôpitaux de Paris, Hôpital Cochin, Paris, France; ^8^ Institut Cochin-Inserm, U1016-CNRS, UMR8104, Paris, France; ^9^ Radiologie, Assistance Publique Hôpitaux de Paris, Hôpital Bichat-Claude Bernard, Paris, France; ^10^ Médecine Nucléaire, Assistance Publique Hôpitaux de Paris, Hôpital Bichat-Claude Bernard, Paris, France; ^11^ AP-HP, Hôpital Bichat, Département Epidémiologie Biostatistiques et Recherche Clinique, F-75018, Paris, France; ^12^ INSERM, Centre d’Investigations cliniques-Epidémiologie Clinique 1425, Hôpital Bichat, F-75018, Paris, France; ^13^ Psychiatrie, Assistance Publique Hôpitaux de Paris, Hôpital Louis Mourier, Paris, France; ^14^ Psychiatrie et Medicine Addictologique, DMU Neurosciences, Assistance Publique Hôpitaux de Paris, GH Saint-Louis-Lariboisiere-Fernand-Widal, Paris, France; ^15^ Néphrologie, Assistance Publique Hô pitaux de Paris, Hô pital Bichat-Claude Bernard, Paris, France

**Keywords:** nephrotoxicity, lithium, CKD-chronic kidney disease, kidney microcysts, bipolar disorder

## Abstract

**Objectives:** Early kidney damage during lithium treatment in bipolar disorder is still hypothetical. We aimed at identifying the determinants of a decreased measured glomerular filtration rate (mGFR) and the accuracy of kidney MRI imaging in its detection.

**Methods:** In this cross-sectional cohort study, 217 consecutive lithium-treated patients underwent mGFR and kidney MRI with half-Fourier turbo spin-echo and Single-shot with long echo time sequences.

**Results:** Median age was 51 [27–62] years, and median lithium treatment duration was 5 [2–14] years. 52% of patients had a stage 2 CKD. In multivariable analysis, the determinants of a lower mGFR were a longer lithium treatment duration (β −0.8 [−1; −0.6] ml/min/1.73 m^2^ GFR decrease for each year of treatment), a higher age (β −0.4 [−0.6; −0.3] ml/min/1.73 m^2^ for each year of age, *p* < 0.001), albuminuria (β −3.97 [−6.6; −1.3], *p* = 0.003), hypertension (β −6.85 [−12.6; −1.1], *p* = 0.02) and hypothyroidism (β −7.1 [−11.7; −2.5], *p* = 0.003). Serum lithium concentration was not associated with mGFR. Renal MRI displayed renal microcyst(s) in 51% of patients, detected as early as 1 year after lithium treatment initiation. mGFR and lithium treatment duration were strongly correlated in patients with microcyst(s) (r = −0.64, *p* < 0.001), but not in patients with no microcysts (r = −0.24, *p* = 0.09). The presence of microcysts was associated with the detection of an mGFR <45 ml/min/1.73 m^2^ (AUC 0.893, *p* < 0.001, sensitivity 80%, specificity 81% for a cut-off value of five microcysts).

**Conclusion:** Lithium treatment duration and hypothyroidism strongly impacted mGFR independently of age, especially in patients with microcysts. MRI might help detect early lithium-induced kidney damage and inform preventive strategies.

## Introduction

Lithium salts are the main prophylactic treatment of bipolar disorder, which is characterized by potentially life-threatening manic and/or depressive episodes. They have proven efficient in the prevention and treatment of acute episodes as well as in the prevention of suicidal risk (Geddes et al., 2004; Investigators et al., 2010; Cipriani et al., 2013; Miura et al., 2014). However, this efficacy is counterbalanced by a narrow therapeutic range that can lead to potentially harmful overdose, and by long-term adverse events (Tabibzadeh et al., 2019). Amongst them, long-term lithium treatment might lead to polyuria and polydipsia, related to impaired urine concentrating ability, and to chronic kidney disease usually with a tubulo-interstitial presentation. The latter is characterized by mild or absent proteinuria (often <1 g/24 h) and cortical and/or medullary small cysts, which supposedly appear at late stages (Grünfeld and Rossier, 2009).

Epidemiological data suggest that chronic use of lithium may lead to impaired glomerular filtration rate (GFR). Indeed, in a retrospective study including 2,500 patients on lithium therapy, Shine et al. showed an increased risk of stage 3 chronic kidney disease (CKD) defined by creatinine-based estimated GFR (eGFR) (Shine et al., 2015). However, in a meta-analysis gathering 385 studies, McKnight et al. showed that eGFR was only 6.2 ml/min/1.73 m^2^ lower in patients treated with lithium salts than in control populations, with an overall small risk of renal failure (McKnight et al., 2012). Another study showed that among 145 patients treated with lithium for more than 15 years, only 21% displayed a measured GFR (mGFR) below the -2 SD cut-off for age-adapted GFR (Bendz et al., 1994). It is thus still unclear whether lithium treatment at therapeutic concentrations is harmful in itself or if the GFR decrease in this population is related to age or to other comorbidities such as metabolic syndrome or hypertension as suggested by the cohort study by Clos et al. (2015). If present, the magnitude of the effect of lithium treatment is also unknown.

The aim of our study was to describe clinical, biological and imaging characteristics of a cohort of lithium-treated patients focusing on renal function and radiological kidney features, and to evaluate the correlates of GFR, including the relationship between kidney function and lithium treatment, using a gold standard method for GFR measurement.

## Methods

### Study Design and Population

From March 2015 to December 2020, 230 consecutive adult patients were referred by psychiatrists to the Department of Renal Physiology for their first visit with nephrologists. Among these patients, we excluded 13 patients who had discontinued lithium treatment, leaving 217 patients who were referred for a systematic check-up. Eligible patients were ≥18 years of age at inclusion, with various durations of lithium treatment, and had neither started dialysis nor underwent kidney transplantation.

The study was performed according to the Declaration of Helsinki. The study was approved by the local ethics committee from APHP. Nord (Institutional Review Board CER-2021-74). All patients provided written informed consent before inclusion in the study cohort.

### Data Collection and Measurements

During a 5 h in-person visit, a large set of clinical and laboratory data were collected, including past medical history, previous use of Non-Steroidal Anti-Inflammatory drugs (NSAIDs) or chemotherapeutic agents, dose and duration of lithium treatment, serum lithium concentration, and current treatment with other psychotropic drugs. Obesity was defined as body mass index (BMI) >30 kg/m^2^. Diabetes was defined as fasting glycemia >7 mmol/L or antidiabetic drug treatment. Hypothyroidism was either defined as a thyroid-stimulating hormone (TSH) level >4.1 mUI/L (above the upper normal limit) or a thyroid hormone therapy. Blood pressure (BP) was the average of three measurements in resting conditions. Hypertension was defined as BP ≥140/90 mmHg or the use of antihypertensive drugs. Patients were instructed to fast (not to eat or drink) from 8 p.m. the day before the admission. Patients were asked to collect 24 h urine the day before admission. Indications to discard first morning void on the first day, and then to collect all urine until the first void the next morning were given by a trained nurse and detailed in a written information document. Fasting blood and urine samples were collected. GFR was measured by urinary clearance of ^51^Cr-EDTA or ^99^Tc-DTPA (GE Healthcare, Velizy, France and Curium, Saclay, France respectively) depending on the time of the visit due to ^51^Cr-EDTA shortage (Vidal-Petiot et al., 2021), determined as the average of 7 consecutive 30 min urinary clearance periods, indexed to the standard body surface area of 1.73 m^2^ as previously described (Froissart et al., 2005). Previous comparison of the two tracers has shown similar results allowing gathering findings from the two techniques (Vidal-Petiot et al., 2021). Plasma lithium concentration was the last available measurement during the last 6 months. Creatinine was measured using an Isotopic Dilution Mass Spectroscopy-standardized enzymatic method.

### Kidney MRI

A renal magnetic resonance imaging (MRI) was proposed to all the patients, and was finally performed in 99 patients due to the remaining patients’ refusal (mainly for claustrophobia or unavailability). MRI was performed with a pre-specified protocol using the following sequences: T2 SSFSE (T2 weighted sequence single-shot fast spin-echo), also called half-Fourier single-shot turbo spin-echo (or HASTE for Siemens), and SSFSE TE long (Single-shot fast spin echo with long echo time). T2 SSFSE is an ultrafast MRI technique with a short acquisition time less susceptible to motion respiratory artifact than other techniques such as echo T2-weighted imaging (Nakayama et al., 2001). SSFSE TE long is a highly T2 weighted sequence allowing an optimal contrast between microcysts and renal parenchyma. This MRI protocol has proven useful in depicting structures containing static fluids including cystic lesions, characterized as T2 hyperintense round areas (Nakayama et al., 2001).

Renal microcysts were defined as small (1–2 mm) round cystic lesions and quantified in both kidneys. As inter and intra-observer reproducibility was poor when counting the total number of microcysts, a semi-quantitative approach was used, and MRI findings were classified as follows: 1/no microcysts, 2/1 to 5 microcysts, 3/6 to 10 microcysts, 4/11 to 20 microcysts, 5/21 to 50 microcysts, 6/51 to 100 microcysts, 7/> 101 microcysts. Images were blindly reviewed and analyzed by 2 senior radiologists and one senior nephrologist. This semi-quantitative scoring was reproducible.

### Statistical Analyses

Categorical variables were described as frequencies and percentages, and continuous variables as median [25th-75th percentiles]. Baseline patients’ characteristics were compared according to mGFR classes (>90, 60–90, <60 ml/min/1.73 m^2^) using Kruskal–Wallis test and Chi-square tests, for quantitative and qualitative variables, respectively. Correlations between mGFR, age and lithium treatment duration (log-transformed) were assessed by Spearman tests. Determinants of mGFR were assessed using multivariable linear regression models. Covariates were selected *a priori* based on potential confounding and included: age, gender, body mass index (<vs. ≥30 kg/m^2^), diabetes, hypertension, hypothyroidism, treatment with other psychotropic drugs, lithium treatment duration in years (log-transformed), lithium galenic formulation (extended or immediate-release formulation), serum lithium concentration and 24 h urinary albumin/creatinine ratio (log-transformed). Interactions between treatment duration and age, gender, and BMI were tested. Normality of the distribution of residuals and homoscedasticity were verified. Multiple imputations using the chained equation method (R package *mice*, *n* = 50 imputed datasets, *m* = 20 iterations) were performed for missing data.

In order to specifically analyze the association between mGFR and renal microcysts, a Spearman correlation was performed between mGFR and lithium treatment duration in patients with microcysts and those without microcysts. A linear regression model was built to explain mGFR according to treatment duration, with an interaction term between treatment duration and renal microcysts to assess the difference in mGFR slopes between patients with and without microcysts. The effect of microcysts on slopes was estimated and a Wald test was performed on the interaction term to compare the two groups.

With an mGFR <45 ml/min/1.73 m^2^ considered as the state of disease, sensitivity (Se) and specificity (Sp) were calculated for each microcyst cut-off score. A Receiver Operating Characteristic (ROC) Curve was plotted in order to measure the Area Under the Curve (AUC) and the Youden Index allowed identifying the most accurate cut-off value for renal microcysts.

A two-sided *p*-value < 0.05 was considered statistically significant. Statistical analyses were conducted using in GraphPad Prism 9.0 and R 3.4 software.

## Results

### Characteristics of the Population

Table 1 summarizes patients’ characteristics. Median age was 51 [27–62] years, and 62% were female. Median BMI was 25.9 [22.9–28.8] kg/m^2^. Median lithium treatment duration was 5 [2–14] years and median dose was 800 [575–1,000] mg/day. The extended-release form was administered in a majority of patients (63%). Median plasma lithium concentration was 0.72 [0.56–0.87] mmol/L. Median mGFR was 78 [63–91] ml/min/1.73 m^2^ with a majority of stage 2 CKD (52%). Microalbuminuria was absent (<3 mg/mmol) in a large majority (83%) of patients and only two patients had a macroalbuminuria (which remained below 0.6 g/24 h). Seven (3%) patients had diabetes mellitus, 26% had hypertension and 36% had hypothyroidism. Among the studied population, three patients were found to occasionally take NSAIDs, with mGFRs of 83, 49.9 and 41.7 ml/min/1.73 m^2^. The use of NSAIDs in the latter patient led to a severe lithium overdose (4.1 mmol/L) resulting in acute kidney injury requiring ICU admission the year before referral in the renal physiology unit.

**TABLE 1:** Characteristics of the patients in the whole cohort and according to measured GFR.

Variables, *n* available data	Total *n* = 217	mGFR >90 ml/min/1.73 m^2^ *n* = 57	60≤ mGFR<90 *n* = 113	mGFR <60 ml/min/1.73 m^2^ *n* = 47	*p*-value
Age, years *n* = 217	51 [37–62]	38 [31–48.5]	51 [28–61.5]	62 [54.5–69]	<0.001
Male patients, *n* = 217	83 (38.2%)	33 (57.9%)	38 (33.3%)	12 (10.5%)	<0.001
Body mass index, kg/m^2^, *n* = 217	25.9 [22.9–28.8]	25.5 [23.1–29.3]	25.6 [22.5–29]	26.8 [23.6–28.8]	0.7
Diabetes mellitus, *n* = 217	7 (3.2)	1 (1.8%)	4 (3.5%)	2 (4.3%)	0.7
Hypertension, *n* = 217	56 (25.8)	6 (10.5%)	27 (23.7%)	23 (48.9%)	<0.001
Hypothyroidism, *n* = 217	78 (35.9)	12 (21.1%)	41 (36%)	23 (53.2)	0.01
Other psychotropic drugs, *n* = 216	152 (70.1%)	35 (61.4%)	83 (73.5%)	34 (72.3%)	0.3
Lithium treatment duration, years, *n* = 217	5 [2–14]	2 (1–5)	5 (2–12)	16 [9.5–30]	<0.001
Extended-release formulation, *n* = 214	138 (63.6%)	41 (71.9%)	74 (64.9%)	23 [48.9%]	0.04
Daily dose, mg/day *n* = 216	800 [575–1,000]	1,000 [788–1,200]	800 [750–1,000]	500 [400–750]	<0.001
Serum lithemia, mmol/L, *n* = 139	0.72 [0.56–0.87]	0.72 [0.53–0.9]	0.75 [0.6–0.9]	0.61 [0.5–0.84]	0.4
mGFR, ml/min/1.73 m^2^ *n* = 217	78 [62.8–90.5]	97.3 [94.1–102.9]	77.3 [71.5–83.4]	46.6 [41–53.7]	<0.001
ACR, mg/mmol creatinine, *n* = 214	1.4 [0.9–2.2]	0.9 [0.8–1.9]	1.4 [1.0–2.0]	2.0 [1.4–4.3]	<0.001
PCR, mg/mmol creatinine, *n* = 216	14.1 [10.1–20.2]	11.4 [8.7–15.3]	14.0 [10.1–19.7]	19.6 [15.6–33.2]	<0.001
Renal MRI findings	*n* = 99	*n* = 28	*n* = 48	*n* = 23	
No microcysts	49 (49.4%)	20 (71.4%)	26 (54.2%)	3 (13%)	<0.001
1–10 microcysts	30 (30.3%)	7 (25%)	15 (31.3%)	8 (34.8%)	<0.001
>10 microcysts	20 (20.2%)	1 (3.6%)	7 (14.6%)	12 (52.2%)	<0.001

Categorical and continuous data are expressed in *n* (%) and in median [quartile 1- quartile 3], respectively. Baseline patients’ characteristics were compared across mGFR, level using the chi-2 test for categorical variables and Kruskal-Wallis Rank Sum Test for quantitative variables. mGFR: measured glomerular filtration rate, ACR: urinary albumin to creatinine ratio, PCR: urinary protein to creatinine ratio, MRI: magnetic resonance imaging.

Compared with patients with mGFR higher than 60 ml/min/1.73 m^2^, patients in the lowest mGFR group (<60 ml/min/1.73 m^2^) were significantly older, with a higher prevalence of hypertension, and were more frequently female. In this population, lithium treatment duration was significantly longer, and daily dose was significantly lower with a lower rate of extended-release lithium preparation. Of note, there was no statistical difference according to GFR groups for serum lithium levels. Even though levels were low in all groups, they also displayed a higher albuminuria and higher proteinuria (Table 1).

**FIGURE 1:**
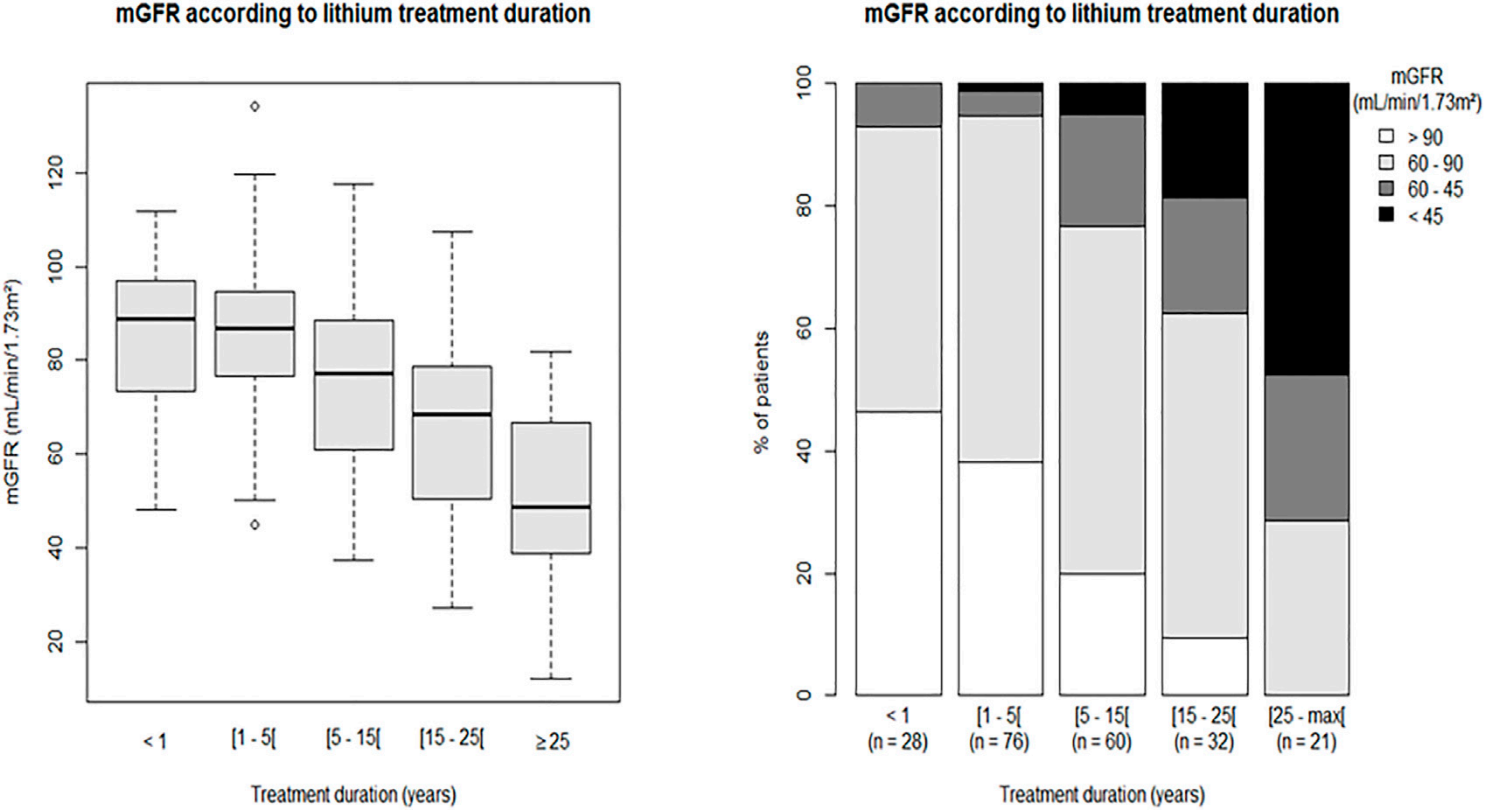
mGFR according to lithium treatment duration. Left panel: median and range of mGFR according to lithium treatment duration. Right panel: stacked bars representing mGFR classes according to lithium treatment duration. mGFR: measured glomerular filtration rate.

The prevalence of CKD was higher in patients with a longer treatment duration (Figure 1). In patients treated for less than 1 year, only two patients had an mGFR <60 ml/min/1.73 m^2^. Among patients treated for more than 25 years, none had a normal mGFR (>90 ml/min/1.73 m^2^).

### Determinants of mGFR

**FIGURE 2:**
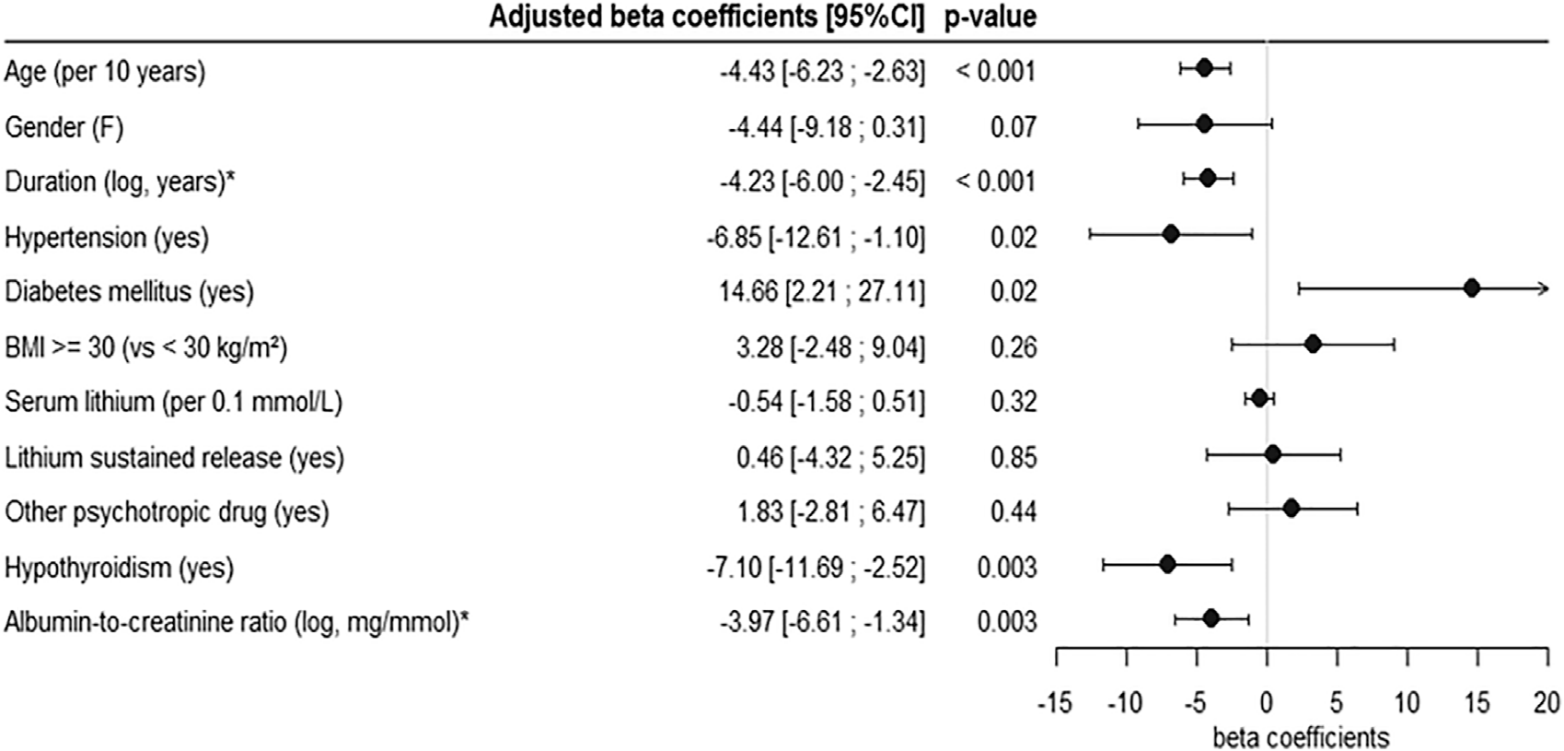
Multivariable analysis of the determinants of mGFR. Determinants were assessed using multivariable linear regression model, with multiple imputations for missing data. All the variables included in the model are represented on the forest plot. F: female, BMI: body mass index, mGFR: measured glomerular filtration rate. * The indicated coefficients correspond to a 2.72-fold [ = exp (1)] increase in lithium treatment duration or in albumin-to-creatinin ratio.

Lithium treatment duration (log-transformed) and age were inversely correlated with mGFR (r = −0.51, *p* < 0.001 and r = −0.54, *p* < 0.001, respectively). In multivariable analysis, lithium treatment duration and age were also independently and negatively associated with mGFR, with a stronger association with lithium treatment duration (β-coefficient: −0.8 [−1; −0.6] ml/min/1.73 m^2^ GFR decrease for each additional year of treatment) compared to age (β-coefficient: −0.4 [−0.6; −0.3] ml/min/1.73 m^2^ for each additional year of age, *p* < 0.001). Association between treatment duration and mGFR did not depend on age or other covariates (non-significant *p*-values for interaction tests) (Figure 2). Albuminuria, hypothyroidism and hypertension were also independently and negatively associated with mGFR (β-coefficients respectively −3.97 [−6.6; −1.3], *p* = 0.003, −7.1 [−11.7; −2.5], *p* = 0.003, −6.85 [−12.6; −1.1], *p* = 0.02). Diabetes was associated with a higher mGFR, but was diagnosed in only seven patients including one with hyperfiltration (defined by an mGFR >120 ml/min/1.73 m^2^). The other tested covariables—including serum lithium concentration, the use of extended-release form of lithium carbonate and treatment with other psychotropic agents (including when specifically analyzing the use of neuroleptic agents, SSRI- selective serotonin reuptake inhibitors-antidepressants, SNRI-serotonin-norepinephrine reuptake inhibitor-antidepressants, MAO-monoamine oxidase-inhibitors)—were not independently associated with mGFR.

### Accuracy of Renal Microcysts for the Diagnosis of Lithium-Related Nephrotoxicity

**FIGURE 3:**
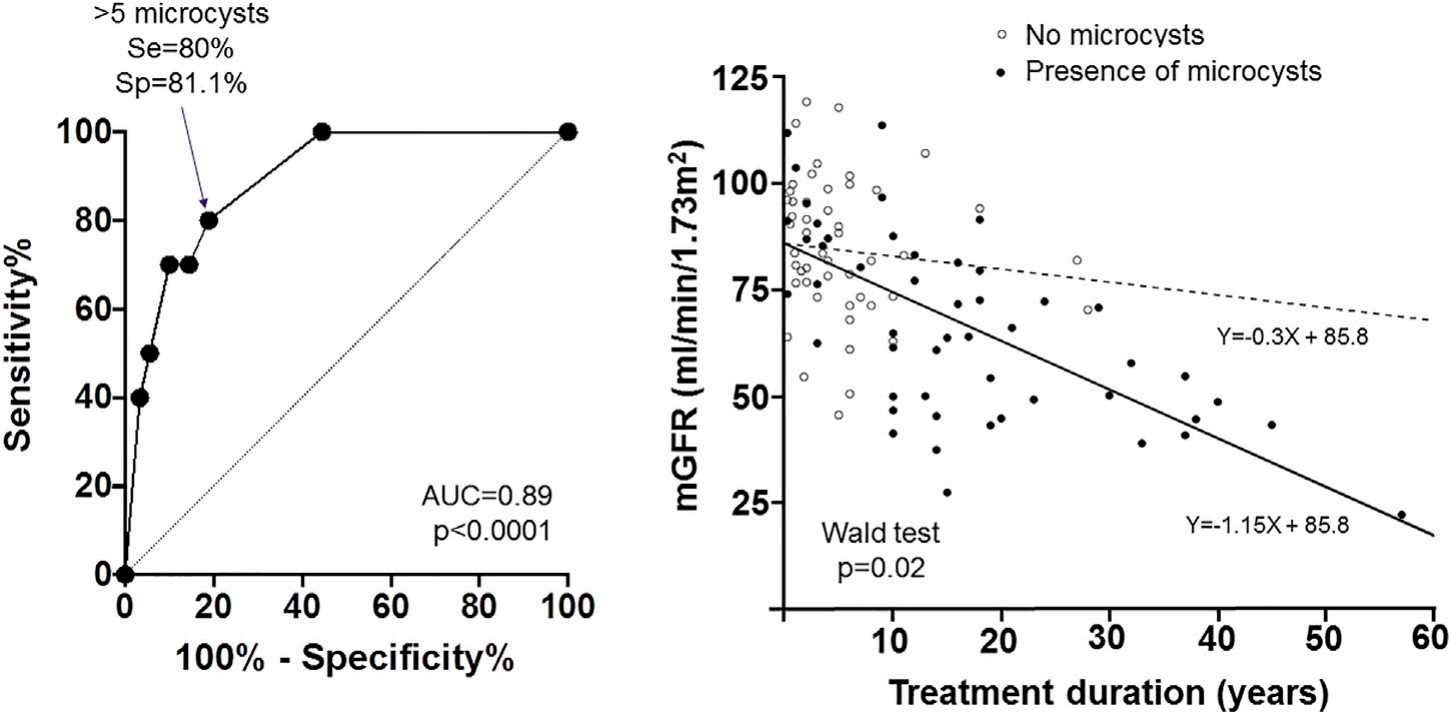
Accuracy of renal microcysts in the diagnosis of lithium-related chronic kidney disease. Left panel: ROC Curve showing the sensitivity and 1—specificity of renal microcysts quantification for the diagnosis of mGFR <45 ml/min/1.73 m^2^. The cut-off value of 5 microcysts had the highest Youden Index, with a sensitivity (Se) of 80% and a specificity (Sp) of 81.1%. Right panel: GFR values according to treatment duration and regression lines in patients with (filled circles and black line) and without (open circles and dashed line) microcysts. A Wald test was performed to test the difference in slopes between the two groups.

Among patients who underwent renal MRI 51% displayed renal microcyst(s), appearing as T2 hyperintense round lesions uniformly and symmetrically distributed throughout the medulla as well as the cortex of normal-sized kidneys. Isolated Bosniak type 1 cysts were also unfrequently observed. Microcysts were present in patients as soon as 1 year after lithium treatment initiation. Patients in the lowest mGFR group had a higher prevalence of renal microcysts. When comparing patients with and without microcysts, mGFR and lithium treatment duration were strongly correlated in patients with microcyst(s) (r = −0.64, *p* < 0.001), whereas no such correlation was found in patients with no microcysts (r = −0.24, *p* = 0.09). This was confirmed by the linear regression of mGFR according to lithium treatment duration in patients with microcysts (slope −1.15, 95% CI [−1.44; −0.86]) and with no microcysts (slope −0.30, 95% CI [−1.03; 0.43]), with a statistically significant difference between the slopes of the two groups (Wald test *p* = 0.02) (Figure 3).

Using receiver operating characteristic (ROC) analysis, we determined the renal microcyst quantification threshold associated with the detection of an mGFR below 45 ml/min/1.73 m^2^ (Figure 3). The AUC was 0.893 (*p* < 0.001). At a cut-off value of 5 microcysts (defined by category 1 = no microcysts, and category 2 = 1 to 5 microcysts), the Youden Index was maximal (0.61), with a Se and a Sp respectively of 80 and 81%.

## Discussion

Our study showed a strong relationship between chronic lithium use and GFR decline, independently of other potential confounding factors. The statistical effect of lithium use on GFR decrease was determined as a 0.8 ml/min/1.73 m^2^ mean decrease in mGFR per year of exposition. Other determinants of a lower mGFR were higher age, higher albuminuria, hypertension and hypothyroidism.

There are conflicting results regarding renal effects of lithium salts (Tabibzadeh et al., 2019). Several reports suggest that even when CKD is present, it is not clinically significant as no increase in the risk of ESKD has been demonstrated (van Melick et al., 2008; Aprahamian et al., 2014; Kessing et al., 2015). In their meta-analysis, McKnight et al. demonstrated a slight eGFR reduction (mean −6.2 ml/min/1.73 m^2^) in patients treated with lithium compared to control groups (McKnight et al., 2012). The main limitation of these studies is the short follow-up duration and the short median treatment duration. In a large retrospective study of laboratory analyses from more than 2,500 patients, Shine et al. observed an increased risk of CKD stage 3 in lithium-treated patients compared to control populations (Shine et al., 2015). In the same line, a French retrospective study showed that 39% of patients aged 20–39 years, and up to 85% in patients aged 70 years or older displayed decreased GFR (defined as an estimated GFR <60 ml/min/1.73 m^2^) (Bassilios et al., 2008). Accordingly, a large Swedish cohort study showed that 1.2% of lithium-treated patients had a serum creatinine level >150 μmol/L (Bendz et al., 2010). As controversial as kidney impairment during chronic lithium use may be, the general view is that lithium treatment induces a slowly progressive tubulo-interstitial nephropathy, that cannot be easily detected in longitudinal studies (Davis et al., 2018).

In this perspective, evolution towards end-stage kidney disease (ESKD) is also poorly defined. A retrospective study among patients on dialysis showed that 0.22% of these patients had a diagnosis of lithium-induced nephropathy (Presne et al., 2003). In the Swedish cohort, the prevalence of ESKD in lithium-treated patients was 0.5%—more than 6 times the estimated prevalence in the general population (Bendz et al., 2010)—after a mean treatment duration of 23 years.

Conflicting results have also been reported in experimental studies regarding lithium effect on kidney (Alsady et al., 2016). As few studies have experimented long-term exposure to lithium, a satisfying experimental model of lithium-induced nephropathy with renal insufficiency is still lacking in order to clarify the renal impact of chronic lithium use.

Our study allowed establishing a clear relationship between lithium use and GFR decrease. Indeed, in this population, the strongest determinant of mGFR was lithium treatment duration. The effect of lithium treatment duration was twice that of age, though a cumulative effect of age and treatment duration might potentiate renal lesions secondary to lithium chronic use on an underpinning senescent renal tissue. Of note, a majority of the patients were treated with the extended-release formulation of lithium carbonate, which is supposed to decrease the risk of kidney disease by limiting the risk of acute rises of serum lithium levels (Turan et al., 2002; Carter et al., 2013; Castro et al., 2016). Noteworthy, a higher serum lithium level was not associated with lower mGFR but the high proportion of missing data (36% of missing serum lithium values) might have limited the statistical power of the study. Our results are in line with the study by Bendz et al. showing no difference in terms of plasma lithium levels between patients with CKD and ESKD and those with normal renal function (Bendz et al., 1994). In contrast, Shine et al. suggested a higher risk of adverse renal outcomes with higher serum lithium levels, but the analysis was not adjusted on baseline eGFR (Shine et al., 2015). In any case, a causal relationship between serum lithium level and mGFR should be interpreted with caution in observational studies, as lower mGFR is also responsible of lower excretion rate of lithium and thus higher serum lithium levels (Alsady et al., 2016). Consistently, in our study, the daily dose of lithium was higher in patients with higher mGFR, which is not surprising as serum lithium level depends on lithium glomerular filtration which guides drug dose adaptation. Moreover, a single plasma lithium measurement is probably not representative of the long-term cumulative exposure.

Age was also an independent determinant of mGFR. Age is a well-demonstrated determinant of GFR decline, with a reported annual decline of 0.3–0.8 ml/min/year depending on the studied population^1^. However, GFR decline over time might reflect kidney senescence rather than pathological processes (Delanaye et al., 2019. The combined effect of age and lithium treatment is likely superior to this physiological GFR decrease over time.

Lithium administration is associated with metabolic disorders including weight gain and thyroid disorders, the pathophysiology of which are not fully understood (Gracious and Meyer, 2005). Obesity is a risk factor for CKD and ESKD (Chang et al., 2018). Other factors influencing BMI such as thyroid disorders (Mariani and Berns, 2012) might also promote kidney disease (Brenner and Meyer, 1982). We thus tested whether obesity or hypothyroidism might determine mGFR in these patients. Our analyses ruled out the role of obesity but found a strong association between hypothyroidism and lower mGFR. As previously reported, hypothyroidism was frequent in our study population (36%). The literature suggests a higher prevalence of hypothyroidism in female patients treated with lithium salts, with no reported effect of treatment duration on that risk. However, to the best of our knowledge, the association between kidney impairment and hypothyroidism in patients treated with lithium had not yet been described. Thyroid hormones exert both direct and indirect effects on renal functions, including cardiovascular effects affecting renal hemodynamics (Mariani and Berns, 2012) potentially explaining the link between hypothyroidism and renal risk reported in previous epidemiological data (Rhee et al., 2015). However, our observational study does not establish causality between hypothyroidism and a lower mGFR, and the hypothesis of an association due to a common pathway of susceptibility to kidney and thyroid toxicities induced by chronic lithium treatment cannot be excluded.

The majority of patients (83%) displayed mild CKD with no albuminuria, and only two patients had a macroalbuminuria (<0.6 g/24 h), suggesting that glomerular disease did not participate in the kidney disease and that CKD was mostly not related to other underlying nephropathies. Yet, albuminuria was associated with a lower mGFR in agreement with current knowledge (Levey et al., 2020). The prevalence of diabetes was also very low (3%) in our cohort, and one patient displayed glomerular hyperfiltration, thus inducing a bias in the association between mGFR and diabetes. Conversely, hypertension was observed in 23% of patients and was an independent determinant of mGFR. It has been shown that CKD both contributes to the development of hypertension, and might result from hypertension (Ku et al., 2019). However, the prevalence of hypertension was far lower in our population compared to previously reported CKD populations (Vidal-Petiot et al., 2018), and even less than the reported prevalence in the general population in France (Perrine et al., 2018). Consequently, our results suggest that though hypertension is a relevant comorbidity contributing to the decrease in mGFR in patients treated with lithium salts, it is probably not a feature of lithium-induced nephropathy.

Renal cysts have been reported during long-term lithium use. However, the majority of published data involve small case series or case reports of patients with numerous microcysts and overt chronic kidney disease (Farres et al., 2003; Golshayan et al., 2012; Judge and Winearls, 2015). It is thus not yet clearly established whether renal microcysts are a specific and early feature of kidney impairment of chronic lithium use and if they might be used as a diagnostic tool. Moreover, data regarding the general population is lacking as usual imaging tools detect cysts of greater size (at least >5 mm) (Rahbari-Oskoui et al., 2014) and previous research reported the incidence of the prevalence of renal cysts (>1 cm) (Simms and Ong, 2014). In our cohort we found that even a relatively small number of microcysts was associated with a decrease in mGFR, with acceptable sensitivity and specificity to detect an mGFR <45 ml/min/1.73 m^2^. The optimal cut-off value was 5 microcysts. Of major importance, while there was a strong negative correlation between mGFR and lithium treatment duration in patients with at least one microcyst, no such association was seen in patients without microcysts, in favor of a strong relationship between mGFR decrease and the presence of microcysts. The presence of microcysts might thus help inform improved strategies such as decreasing lithium exposure and preventing other comorbidities and nephrotoxic agents. These results must however be interpreted with caution due to the sample size (*n* = 99). Further investigation is also needed to establish if they reflect the degree of irreversibility, as the potential benefit of treatment discontinuation is still poorly known (Bendz et al., 1994; Markowitz et al., 2000), and shall be weighed against the established suicidal risk in this setting (Baldessarini et al., 1999).

Our study displays some limitations. Regarding lithium treatment, serum lithium level was not measured the same day as renal evaluation. However, our patients were stable, and underwent regular follow-up by psychiatrists. Second, neither information regarding cumulative lithium dose nor episodes of overdose were available. We were thus not able to investigate whether nephrotoxic effect of lithium was related to acute serum lithium level rises, or to a cumulative effect of low dose exposition (Carter et al., 2013). Of note, previous data suggest that renal impairment during chronic lithium use is not related to cumulative lithium dose (Bendz et al., 1994). Finally, the cross-sectional design of the study prevents us from analyzing the predictive value of lithium treatment duration on mGFR decline. Also, we cannot exclude that lithium was initiated in some patients with an already altered GFR, but it can be noted that only 2 out of 42 patients treated with lithium for less than 1 year had an mGFR below 60 ml/min/1.73 m^2^.

In conclusion, our study confirmed the independent effect of lithium exposure on kidney function. This effect combined with that of age, hypothyroidism, hypertension and microalbuminuria on mGFR demonstrates that a close monitoring is necessary including blood pressure measurements, GFR estimation, albuminuria and thyroid hormone levels. MRI might also be considered as a useful tool in order to detect microcysts even during early stages of treatment. The association of a lower GFR with microcysts and hypothyroidism questions the issue of a differential individual genetic or acquired susceptibility to lithium treatment. These data might help inform improved strategies to prevent possibly irreversible kidney damage during chronic lithium use.

## Data Availability

Data regarding this research is available upon request to the corresponding author.

## Footnotes

1 Chapter 2: Definition, identification, and prediction of CKD progression. Kidney Int Suppl. 2013;3(1):63–72. doi:10.1038/kisup.2012.65
